# Encapsulation of Gemcitabine on Porphyrin Aluminum Metal-Organic Framework by Mechano-Chemistry, Delayed Drug Release and Cytotoxicity to Pancreatic Cancer PANC-1 Cells

**DOI:** 10.3390/molecules29133189

**Published:** 2024-07-04

**Authors:** Sheriff Umar, Alexander Samokhvalov

**Affiliations:** Department of Chemistry, Morgan State University, 1700 East Cold Spring Lane, Baltimore, MD 21251, USA

**Keywords:** gemcitabine, mechano-chemistry, metal-organic framework, drug release, pancreatic cancer cells

## Abstract

Gemcitabine is a widely used antimetabolite drug of pyrimidine structure, which can exist as a free-base molecular form (Gem). The encapsulated forms of medicinal drugs are of interest for delayed and local drug release. We utilized, for the first time, a novel approach of mechano-chemistry by liquid-assisted grinding (LAG) to encapsulate Gem on a “matrix” of porphyrin aluminum metal-organic framework Al-MOF-TCPPH_2_ (compound **2**). The chemical bonding of Gem to compound **2** was studied by ATR-FTIR spectroscopy and powder XRD. The interaction involves the C=O group of Gem molecules, which indicates the formation of the encapsulation complex in the obtained composite. Further, the delayed release of Gem from the composite was studied to phosphate buffered saline (PBS) at 37 °C using an automated drug dissolution apparatus equipped with an autosampler. The concentration of the released drug was determined by HPLC-UV analysis. The composite shows delayed release of Gem due to the bonded form and constant concentration thereafter, while pure Gem shows quick dissolution in less than 45 min. Delayed release of Gem drug from the composite follows the kinetic pseudo-first-order rate law. Further, for the first time, the mechanism of delayed release of Gem was assessed by the variable stirring speed of drug release media, and kinetic rate constant *k* was found to decrease when stirring speed is decreased (diffusion control). Finally, the prolonged time scale of toxicity of Gem to pancreatic cancer PANC-1 cells was studied by continuous measurements of proliferation (growth) for 6 days, using the xCELLigence real-time cell analyzer (RTCA), for the composite vs. pure drug, and their differences indicate delayed drug release. Aluminum metal-organic frameworks are new and promising materials for the encapsulation of gemcitabine and related small-molecule antimetabolites for controlled delayed drug release and potential use in drug-eluting implants.

## 1. Introduction

Each year, cancer is diagnosed in over a million patients in the United States, and over half a million people die from it. Pancreatic cancer is highly lethal and difficult to cure, and conventional chemotherapy is conducted by systemic (intravenous or oral) drug administration. Gemcitabine is a widely used antimetabolite of pyrimidine structure [1] and the first-line chemotherapy agent against several cancers. The FDA approved gemcitabine for the systemic (intravenous) chemotherapy of pancreatic and a few other cancers, and this drug is being extensively studied [2]. One limitation of systemic administration is the “burst” effect—a quick increase in drug concentration. The active time window of the drug is limited (minutes to a few hours) due to metabolization, while the amount of the administered drug can be large (up to a few grams). Hence, the side effects due to the “burst” can be acute, particularly in long-term treatments, e.g., post-surgical maintenance [3].

An alternative drug administration can be based on time-delayed and/or local release by drug-eluting implants [4]. The number of available implants and choices of materials for cancer chemotherapy via delayed release are limited. The only FDA-approved anti-cancer drug-eluting implant is the Gliadel wafer for treating glioma, which contains carmustine (drug) and organic polymer (matrix).

The matrices for the encapsulation of drugs, including antimetabolites, are biopolymers, synthetic polymers, vesicles and nanoparticles. Metal-organic frameworks (MOFs) are porous nanostructured coordination polymers, which emerged about a decade ago [5] as a highly efficient matrix for delayed drug release. MOFs consist of metal cations and anions of organic linkers, which can form bonds with drug molecules [6] and cause delayed drug release by desorption to the release media (buffer solution) or cell culture. Aluminum MOFs (Al-MOFs) [7] have high chemical stability and low toxicity, contain linkers of variable structure for binding drug molecules and are insoluble in water which makes them suitable as matrices of drug-eluting implants. In contrast, the majority of studies of delayed drug release were conducted with colloidal MOFs, e.g., [8].

Usually, the encapsulation of drugs to matrices is conducted by sorption in the solution of the drug [6]. However, most antimetabolite drugs of pyrimidine structure, including gemcitabine, are poorly soluble in any solvent; hence, novel methods of drug encapsulation are needed. Mechano-chemistry studies reactions of solids under mechanical impact; these reactions cover organic and inorganic chemistry, and they are used in research and industrial processing of pharmaceuticals [9]. The mechano-chemical process conducted using grinding fluid [10] is denoted as liquid-assisted grinding, LAG. In the mechano-chemical process, the transfer of energy occurs, and solids are “activated” [11] which may lead to unusual reactions. To our knowledge, mechano-chemical reactions of MOFs with drugs have not been reported.

Vibrational spectroscopy is commonly used in the analysis of pharmaceuticals and, in particular, those prepared or processed by grinding [9]. The attenuated total reflectance Fourier transform infrared (ATR-FTIR) spectroscopy was utilized in the characterization of pharmaceuticals [12] including milled drugs [13] and the identification of bonds in them.

Delayed release of medical drugs, and specifically anti-cancer drugs, is of significant interest. In most studies, release occurs from the composite “drug/matrix” to the buffer solution, such as phosphate-buffered saline PBS [14] at 37 °C in the dissolution vessel. Periodically, a small aliquot is withdrawn for analysis, and the obtained curve (e.g., drug concentration vs. time [15]) is termed “drug release profile”. However, when aliquots are collected manually [16], this creates operator errors and does not allow for collecting many points in time (e.g., overnight). Further, the quantitative analysis of released drugs is often conducted by UV-Vis spectrophotometers or, preferably, HPLC-UV instruments.

Here, the automated drug dissolution apparatuses would be very convenient, since they have the autosampler modules where the samples are collected, and samples are ready for the subsequent HPLC analysis (only nano-filtration is needed). The automated drug dissolution apparatuses have been utilized for dissolution studies of formulations in the physical form of pellets (tablets), e.g., [17], and powders (microspheres), e.g., [18]. To our knowledge, automated drug dissolution apparatuses have not been utilized in studies of the release of any drugs from any MOFs.

Studies of the toxicity of chemical agents to live cells are common in research on delayed drug release, e.g., [8,16]. The cytotoxicity of agents is often determined by photometric (colorimetric) methods [19], which lead to cell death, and tests are limited by 72 h. When continuous testing of cellular response and longer (>72 h) time is needed, the real-time cell analysis, RTCA [20], is very useful. It can be conducted by an xCELLigence RTCA system, which periodically measures electric impedance in the cell culture without affecting the viability of cells [21], and the cell index (CI) is a measure of the number and size of live cells.

Herein, we report the following. First, this is the encapsulation of the gemcitabine free base (Gem) on aluminum porphyrin MOF compound **2** as a matrix (Figure S1) conducted by LAG.

Second, the obtained composite was characterized by ATR-FTIR spectroscopy and powder XRD to understand the mechanism of encapsulation of Gem. Third, the obtained composite was tested in the delayed release of gemcitabine to PBS as a drug release medium at 37 °C, in comparison to pure Gem. Here, the automated dissolution and sampling system was used for the first time, and analysis of the concentration of released Gem was conducted by an HPLC-UV method. Fourth, the mechanism of drug release was assessed using variable speed of agitation in the drug release vessel. Fifth, formal chemical kinetic analysis was conducted to obtain the rate law and rate constants of drug release. Finally, testing of the time-resolved response of pancreatic cancer PANC-1 cells to the composite vs. pure Gem was conducted by the xCELLigence RTA system in a timescale of up to 6 days.

## 2. Results and Discussion

### 2.1. Instrumental Characterization of Gem (Drug) and Compound ***2*** (Matrix)

**Figure 1:**
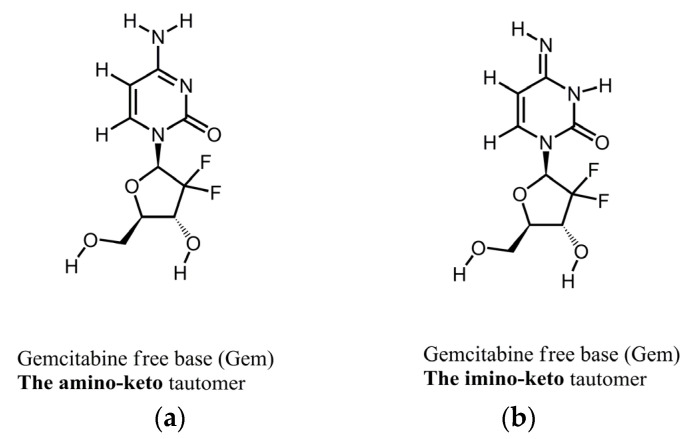
Molecular structures of tautomers of Gem. (**a**) The amino-keto; (**b**) the imino-keto.

Figure 1 shows the molecular structures of the reported tautomeric forms of Gem, and Figure S2 shows its ATR-FTIR spectra.

The spectrum in Figure S2 is consistent with the spectrum of Form 1 of Gem as reported by us previously [22]. Namely, this is a mixture of the amino-keto and the imino-keto tautomers of Gem. The assignments of the most characteristic peaks [22] are in Table 1, and they are marked in Figure S2.

Further, the Gem drug and compound **2** matrix have a rather complex chemical structure and many IR spectral peaks. To explore their interaction, it is first necessary to identify the non-overlapping spectral ranges and the most characteristic peaks in the FTIR spectra of Gem and compound **2**.

Figure S3 shows the FTIR spectra of compound **2**, plotted in the same ranges as for Gem in Figure S2; peak assignments were reported previously [23]. Few IR peaks of Gem (see Figure S2 and Table 1), such as vibrations of the carbonyl group C=O, do not overlap with peaks of compound **2**. These spectral peaks can be monitored before and after the LAG procedure to learn about the interaction of Gem and compound **2**.

### 2.2. The Behavior of Gem and Compound ***2*** in the LAG, When Present Separately

**Figure 2:**
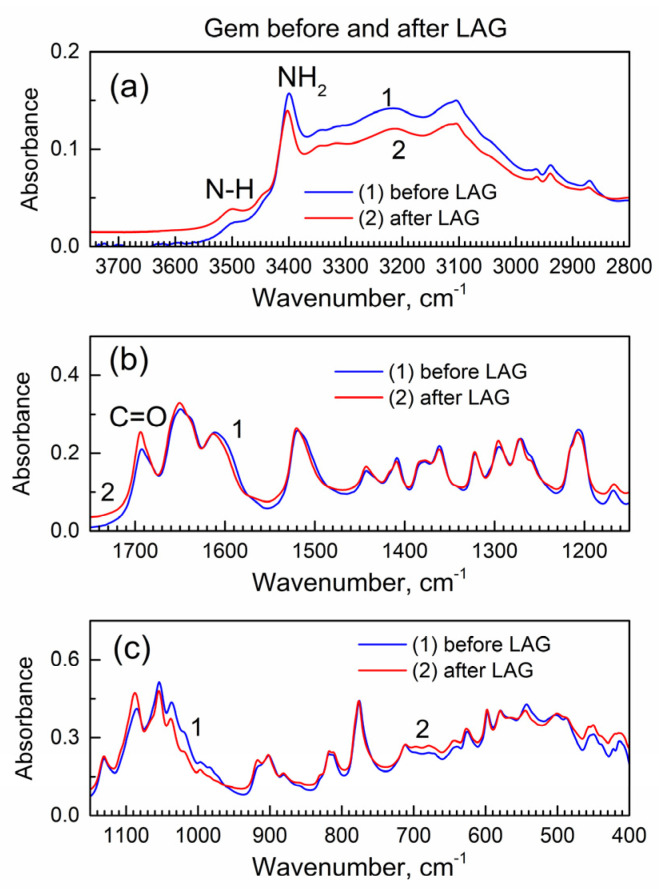
The ATR-FTIR spectra of Gem before and after LAG. (**a**) High wavenumber range; (**b**) mid-IR range; (**c**) low wavenumber range.

The LAG procedure is known to often result in chemical reactions of the involved compounds. Hence, before analysis of the interaction of Gem and compound **2** during the LAG, it is important to learn about the possible reactions of each compound, when present individually, during the LAG under the same conditions. Figure 2 shows the IR spectra of Gem before and after LAG.

Importantly, there are no changes in the spectra which indicates that Gem (when present alone) does not undergo (a) decomposition under grinding, (b) tautomerization or (c) the formation of bonds with grinding fluid (isopropanol). The interaction of Gem with isopropanol (see its vibrational spectrum in [24]) would result in the inclusion of the latter and the presence of additional peaks due to the C-H groups at ca. 2800–3000 cm^−1^ and the O-H peak at ca. 3500 cm^−1^, and this is not the case.

Figure S4 shows the powder XRD patterns of Gem before and after LAG (the patterns were offset by a Y axis for better visibility). It is consistent with the literature XRD pattern of Gem [25] that has the orthorhombic lattice of the Pmna space group, and the prominent reflections are the (002) at 9.5 deg., the (301) at 15.8 deg., the (210) at 16.2 deg., the (303) at 20.8 deg. and the (122) at 27.7 deg.; the lattice parameters are *a* = 17.641(8) Å, *b* = 6.985(1) Å and *c* = 18.653(2) Å. The numeric peak fitting of the sharp, high-intensity (122) peak of Gem at 2θ = 27.9 deg. in Figure S4 was conducted with the Gaussian function. Then, Scherrer’s analysis was used to determine the average nanocrystal size. Here, the equation D = *k* λ/β cos(θ) was used where *k* is a constant (the shape factor with numeric value 1.075 for spherical nanoparticles [26]), λ is an X-ray wavelength, β is the full-width at the half-maximum (FWHM) of the diffraction peak (in radian), and θ is the Bragg angle. This yields an average nanocrystal size of Gem at ca. 30 nm, both before and after LAG, consistently with the absence of changes after LAG as in the FTIR spectra in Figure 2.

**Figure 3:**
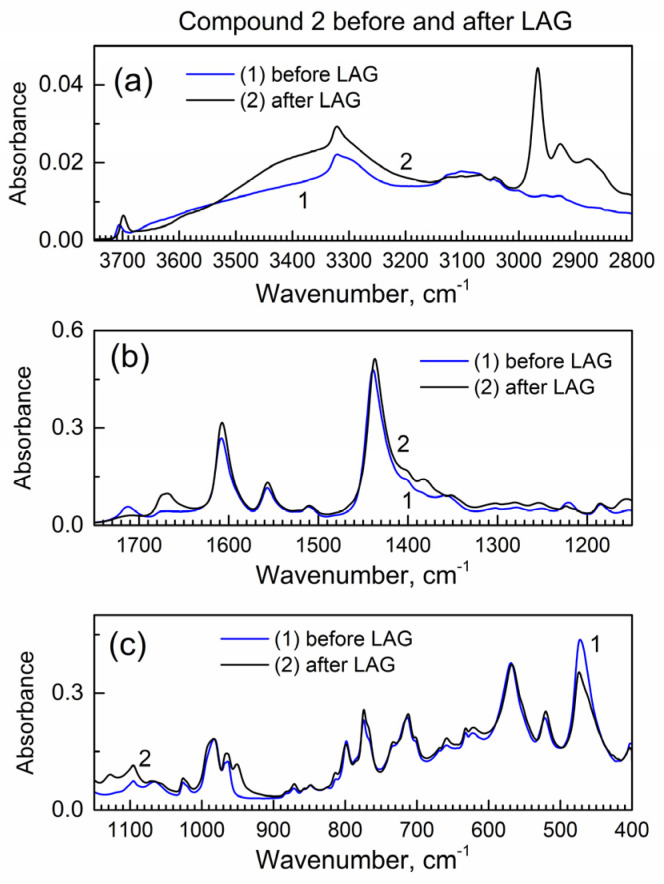
The ATR-FTIR spectra of compound **2** before and after LAG. (**a**) High wavenumber range; (**b**) mid-IR range; (**c**) low wavenumber range.

Next, we studied how drug encapsulation “matrix” compound **2** behaves in the LAG (Figure 3); the IR peaks remain the same which indicates no decomposition during LAG. In Figure 3a, the new peak at ca. 2960 cm^−1^ is apparently due to the inclusion of isopropanol grinding fluid (see its vibrational spectrum in [24]) to compound **2**, and it corresponds to the C-H bond vibrations.

Figure S5 shows the powder XRD pattern of compound **2** before and after LAG. There is the same set of peaks which indicates that no decomposition of compound **2** occurred. However, the major peak at ca. 7.6 deg is wider after LAG. Upon the Scherrer analysis of this peak, the nanocrystal size is 57 nm before LAG and 23 nm after LAG; the smaller nanocrystal size after LAG is consistent with grinding compound **2**.

### 2.3. The Effect of Compound ***2*** on Gem in the LAG Procedure

We analyzed the IR spectra of Gem after the LAG procedure when (a) compound **2** is absent and (b) when it is present (forming the composite) (Figure S6).

Several spectral IR ranges of Gem are “blocked” by strong peaks of compound **2**, so only the non-overlapped IR peaks of Gem (Table 1) were analyzed. For most peaks of Gem, the IR absorbance is decreased in the composite after LAG (the “dilution effect” by the matrix). The carbonyl C=O group in Gem is one of its major markers in IR spectra, considering that the tautomerization of Gem significantly changes the shape of the corresponding IR peak [22]. In Figure S6, after LAG, there is a shift of peak due to the C=O group in Gem in composite vs. pure Gem; this indicates the bonding of Gem to compound **2**.

**Figure 4:**
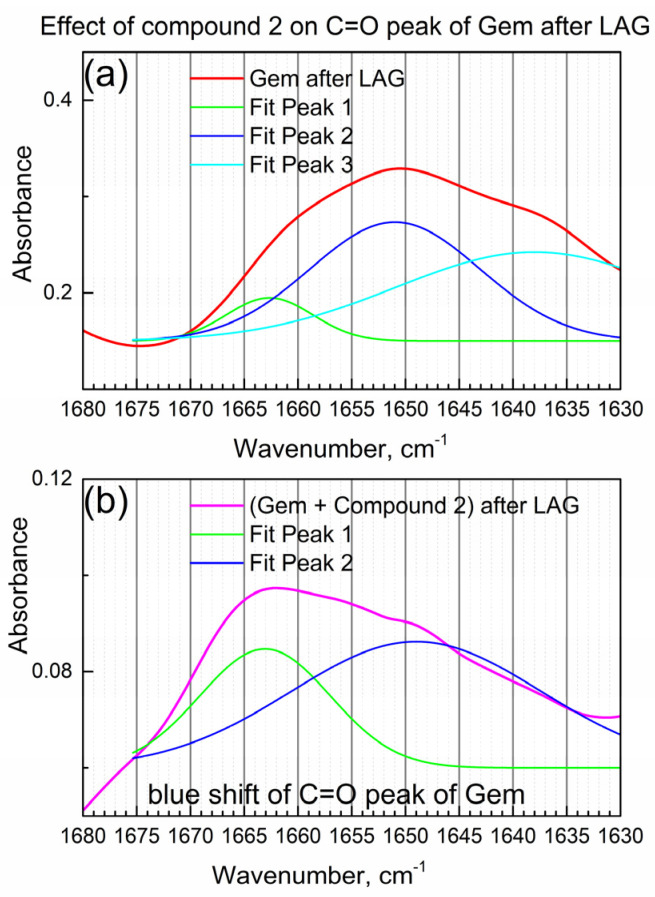
The effect of compound **2** on the C=O peak of Gem. (**a**) Gem after LAG; (**b**) Gem + compound **2** after LAG.

Figure 4a shows the detailed view of the IR peak due to the C=O bond in Gem after LAG when compound **2** was not present.

In Figure 4a, the IR “triplet” peak (the center peak and two shoulders) of the C=O group in Gem was numerically fitted by the triple Gaussian function. Peak maxima are at 1662, 1650 and 1638 cm^−1^ consistently with the “triplet” structure of the C=O peak of the imino-keto tautomer of Gem [22]. On the other hand, the amino-keto tautomer, which is also present in Gem in this work (see Table 1), has one narrow peak of the C=O bond at about the same wavenumber, so it is not readily observed.

Figure 4b shows the IR peak due to the C=O group of Gem after the LAG in the presence of compound **2** (i.e., in the composite). Its shape is significantly modified, and an apparent spectral “blue shift” (shift to higher wavenumbers) is observed which indicates the interaction of the C=O group of Gem with compound **2**. The significant change in the IR peak of the C=O group of Gem after LAG with the compound is confirmed by numeric fitting (see Figure 4b). The shoulder at 1663 cm^−1^ is relatively higher, while the shoulder at 1649 cm^−1^ is lower (compare Figure 4a,b), consistently with the interaction of Gem with compound **2** and possibly partial tautomerization.

An interesting question is whether all or part of Gem is involved in the interaction with compound **2**. Figure S7 shows the effect of compound **2** on the powder XRD pattern of Gem. First, the characteristic XRD peak of Gem is the one that does not overlap with the XRD peaks of compound **2** (see Figure S5): this is the (002) peak at ca. 9.5 deg. in Figure S7a. Figure S7b,c show numeric fittings of the highest (002) XRD peak of Gem for the Scherrer analysis of nanocrystal size. For Gem in the composite (Figure S7c), the (002) peak is much wider than for Gem alone after LAG (Figure S7b) which indicates the lesser degree of crystallinity of Gem when compound **2** was present. Namely, for Gem alone after LAG, the average nanocrystal size is 36 nm, and for Gem in the composite after LAG, it has decreased to ca. 16 nm. This indicates significant dispersion of Gem after LAG in the presence of compound **2**, due to the interaction with compound **2**.

On the other hand, the presence of the individual XRD peaks of Gem indicates that only a fraction of the total amount of Gem is encapsulated, and a certain amount of free (non-bonded) Gem exists in the composite. Hence, the powdered product of the interaction of Gem and compound **2** in the LAG consists of two compounds. First, this is the “encapsulation complex” formed as a result of LAG with the tentative formula Gem_x_MOF_y_ (where MOF is compound **2**). In the initially loaded equimolar mixture of Gem and compound **2**, not all Gem was encapsulated. Second, this is the not encapsulated (free) Gem.

The formation of the encapsulation complex was inferred from complementary data by ATR-FTIR spectroscopy and powder XRD. Below, we test the presence of the encapsulation complex by studies of the delayed release of Gem.

### 2.4. Delayed Release of Gem to PBS from the Composite with Compound ***2***

The reported studies of encapsulation and delayed release of gemcitabine on MOFs are rare and limited to iron MOFs. Rodriguez-Ruiz et al. [8] reported the encapsulation of gemcitabine monophosphate (Gem-MP) on the nanoMOF variety of MIL-100(Fe), by mixing solutions of Gem-MP and nanoMOFs MIL-100(Fe). The release of Gem-MP to the PBS media [8] was fast with the “burst effect”. To our knowledge, there are no studies of encapsulation and delayed release of the gemcitabine free base or its derivatives, on any MOFs in a physical form other than nano-colloids. Additionally, there are no studies on the encapsulation of gemcitabine or its derivatives by any method other than sorption in solution.

First, we analyze the temporal profile of the release of Gem to PBS at 37 °C under conditions of accelerated drug release. Namely, this was at the fast mechanical stirring of 200 rotations per minute (rpm) of a paddle in a drug release vessel (see Section 3). The molar concentration of Gem in drug release media was determined using the calibration plot of HPLC-UV analysis (Figure S8).

**Figure 5:**
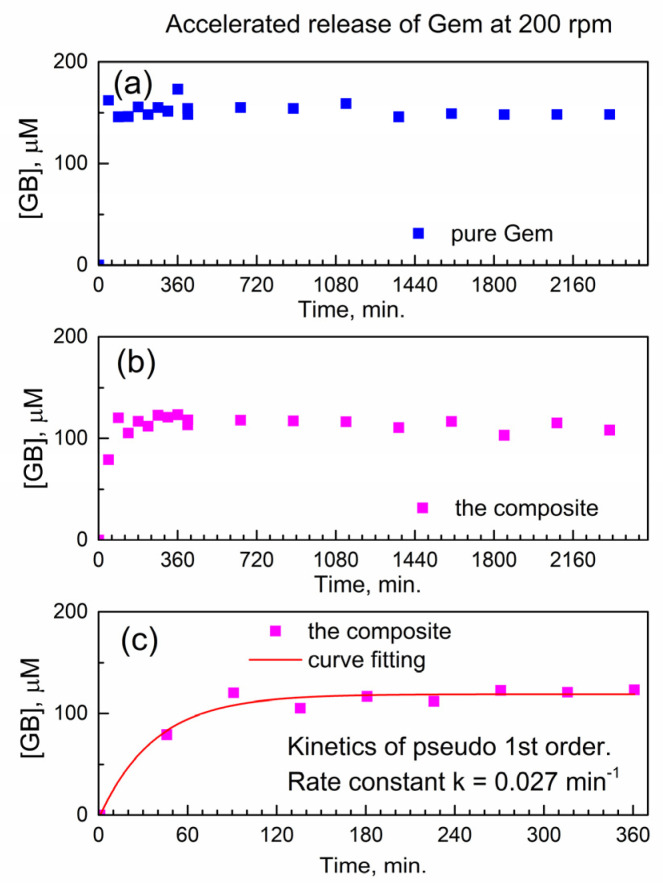
Temporal release profile of Gem to PBS at 37 °C under accelerated conditions (200 rpm). (**a**) Pure Gem; (**b**) the composite; (**c**) fitting the kinetics of Gem release from the composite.

Figure 5a shows the temporal profile of the dissolution of pure Gem in PBS as the molar concentration of the drug in the release buffer vs. time. As expected, the pure drug quickly and fully dissolves within <45 min. (the “burst effect”), and subsequently, its molar concentration remains constant.

Figure 5b shows the temporal profile of the release of Gem from the composite with compound **2**. Here, delayed release is observed without the “burst” and with a gradual increase of [Gem] followed by the plateau. This indicates delayed release of Gem from the encapsulation complex Gem_x_MOF_y_. This finding is of importance since the constant concentration of the released drug in solution represents the therapeutically desired “time window” (with drug concentration being constant in time).

Additionally, the achieved “concentration window” of released Gem in Figure 5b is close to its highest molar concentration 100 μM reported in the cytotoxicity tests with PANC-1 cancer cells [27]. After the delayed release of Gem, the PBS as drug release media contains only Gem and the “linker” of compound **2** (porphyrin TCPPH_2_) which implies partial hydrolysis of the encapsulation matrix.

This is of interest to understand how the chemical kinetics of delayed drug release would allow predicting the concentration of the drug at the given time interval. To our knowledge, there is no kinetic analysis of delayed release of gemcitabine or its derivatives from any MOF.

Figure 5c shows a numeric curve fitting of the drug release profile, by using the kinetics of the pseudo-first-rate law [28]. Here, the concentration of the product, molar concentration [Gem] in the release media, is given as y(t) = A + B × (1 − exp(−*k* × t)). In this formula, the *k* is an effective kinetic rate constant, B is the proportionality coefficient, and A is an offset. The kinetic curve in Figure 5c is well modeled by the pseudo-first-order rate law, with a good value of adjusted goodness-of-fit parameter R^2^_adj_ = 0.96. Since the composite is used in the physical form of powder, the rate constant *k* is likely affected by the diffusion of Gem molecules inside the powder and/or drug release media. Therefore, the numeric value of the effective kinetic rate constant is *k*_eff_(200) = 0.02671 ± 0.00483 min^−1^ where “200” represents the stirring speed (in rpm) in the accelerated conditions of drug release.

**Figure 6:**
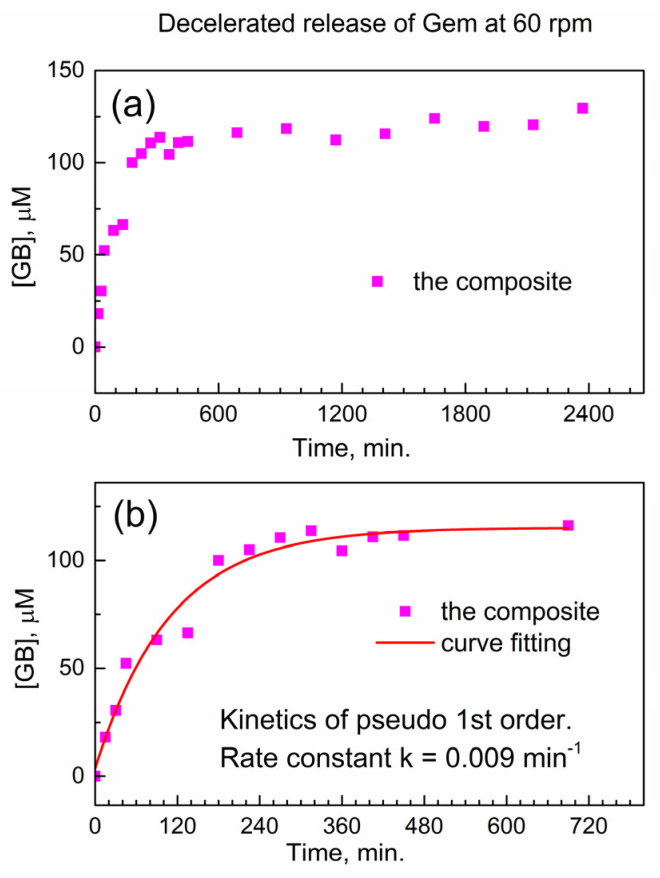
Delayed release of Gem from the composite to PBS at 37 °C under decelerated conditions (60 rpm). (**a**) Temporal profile; (**b**) numeric fitting of kinetics of delayed Gem release.

We test this hypothesis in Figure 6 below when the stirring speed in the drug release vessel is decreased. In Figure 6a, the release of Gem is shown at decelerated conditions (decreased stirring speed of 60 rpm), and the delayed release of Gem continues up to ca. 1800 min. In Figure 6b, the initial stage of the drug release profile was modeled with the kinetic pseudo-first-order rate law, and the effective kinetic rate constant of the decelerated drug release is *k*_eff_(60) = 0.00914 ± 0.00135 min^−1^. The ratio between this value and the effective rate constant of the accelerated release *k*_eff_(200) = 0.02671 ± 0.00483 min^−1^ is equal to about 1/3. This is close to the ratio of stirring speeds in the dissolution vessel (60 rpm versus 200 rpm); hence, the delayed release of Gem from the encapsulation complex is governed by diffusion. This finding is important in the context of potentially using the reported composite for delayed drug release from drug-eluting implants.

Namely, this study is the first report of (a) the encapsulation of Gem on any MOF (and any other matrix) by the mechano-chemical method, (b) using the automatic dissolution and sampling station in studies of its delayed release and (c) the kinetics of delayed release of the so-encapsulated Gem. In the live tissue and cells, mass transfer (i.e., spontaneous diffusion of the drug) is much slower than in the reported experiments of forced agitation in drug release vessel. This allows the expectation of a much longer delayed release of Gem under in vivo conditions and in vitro experiments with cell culture (see below).

### 2.5. The Real-Time Prolonged Cytotoxicity Assay of PANC-1 Cells Using xCELLigence Instrument

**Figure 7:**
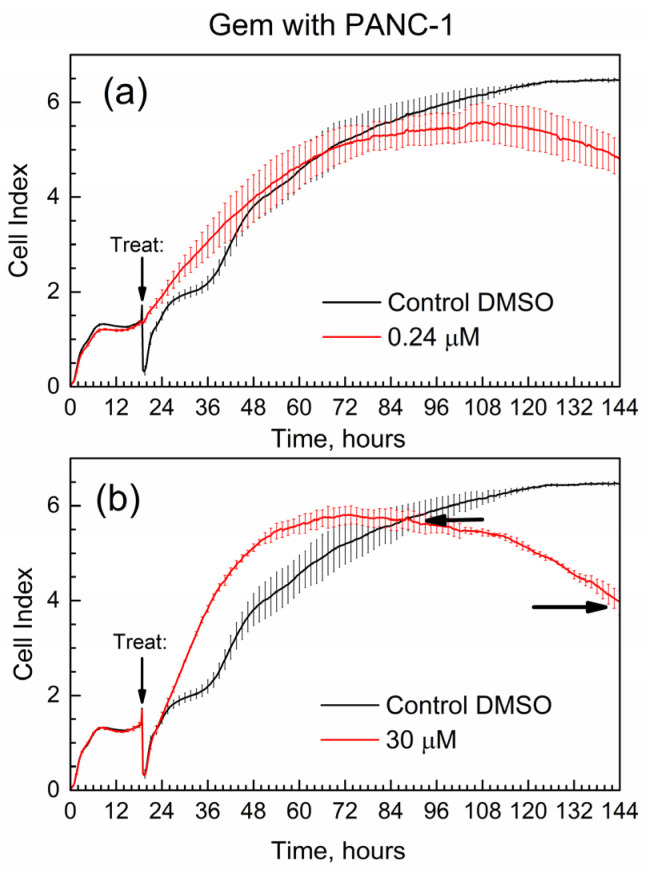
Time-dependent cell index of PANC-1 cells treated with Gem by xCELLigence growth assay. (**a**) 0.24 μM; (**b**) 30 μM.

Figure 7 shows the cell index (CI) of the time-dependent growth of PANC-1 cells treated with pure Gem; the time of addition of the drug (“treat” time) is marked with an arrow. Cell media with added DMSO was used in all E-wells of the xCELLigence experiments for the below data, and it was selected as control in Figure 7.

First, the data in Figure 7 are overall consistent with the known toxicity of gemcitabine to PANC-1 cells: at high drug concentration, the number of surviving cells (the CI) is lower by the end of the experiment. Second, the data are supported by the well-known drug resistance of PANC-1 cells: cell survival (assessed via the CI) is not zero even at the high concentration of gemcitabine [27] at 30 μM. One of the quantitative metrics in the RTCA experiments is the slope of the CI in time, with units of measure 1/h [29]. Here, we used similar metrics; in Figure 7b, horizontal arrows indicate the time points when the cell index of the drug becomes equal to that of control (CI-1 at time t1) and at the end of the test (CI-2 at time t2). The slope for gemcitabine slope(GB) = (C2-1 − CI-1)/(t2 − t1) = −0.033 1/h. The negative numeric value of slope(GB) is indicative of how quickly the suppression of cell growth occurs in time.

**Figure 8:**
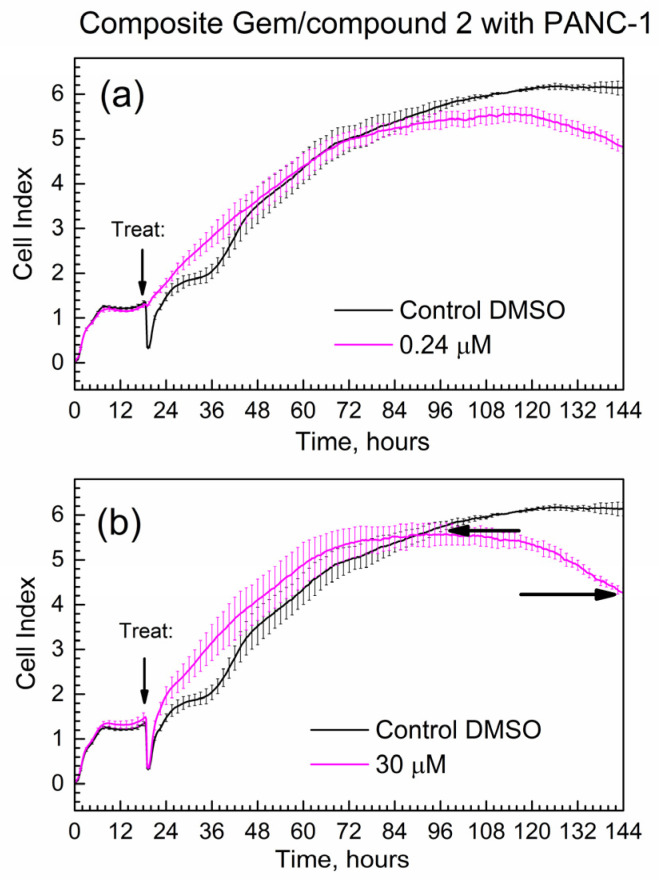
Time-dependent cell index of PANC-1 cells treated with the composite that contains Gem by xCELLigence growth assay. (**a**) 0.24 μM; (**b**) 30 μM.

Figure 8 shows the time-dependent CI of PANC-1 cells treated with the composite; mass loadings were calculated to contain the same mass of Gem as for Figure 7. Overall, the progression of the CI in time is similar for pure Gem and the composite, while the slope(comp) = −0.024 1/h.

Importantly, the absolute numeric value of the slope of the composite at 0.024 1/h is less than the absolute numeric value of the slope of gemcitabine at 0.033 1/h. This difference at ca. 30% is significantly larger than the % errors of CI at start and end time points (<10% error). This indicates that the Gem drug in the composite is released and acts slower compared to the pure drug. It is consistent with the delayed release of Gem from the composite to PBS in kinetic tests in Figure 5 and Figure 6.

## 3. Materials and Methods

### 3.1. Chemicals

Gemcitabine free base (Gem) was obtained from TCI America (product # G0544, Portland, OR, USA) of 98.0% purity (by HPLC) and stored in a freezer at −80 °C after receiving. For synthesis of encapsulation “matrix” compound **2** (actAl-MOF-TCPPH_2_), the first precursor was tetrakis(4-carboxyphenyl)porphyrin (abbreviated TCPPH_2_) of ≥97.0% purity (from TCI America). The second precursor was aluminum chloride AlCl_3_·6H_2_O of 99% purity (from Alfa Aesar, Ward Hill, MA, USA). For synthesis and purification of compound **2**, the solvents were N,N-dimethylformamide (DMF) of ≥99.5% purity (from TCI America) and acetone of the ACS purity of 99.5+% (from Thermo Scientific Chemicals, Waltham, MA, USA). For the LAG, the grinding fluid was isopropanol of 99% purity (from Ward’s Science, Rochester, NY, USA).

### 3.2. Synthesis of the Activated MOF (Matrix) actAl-MOF-TCPPH_2_ (Compound ***2***)

This compound was prepared and activated as reported by us earlier [23]. Briefly, first, the non-activated form of this MOF (asisAl-MOF-TCPPH_2_) was synthesized by an autoclave method. Next, to remove the volatile impurities, the asisAl-MOF-TCPPH_2_ was activated at 200 °C in the vacuum oven for 21 h, and the obtained compound **2** was promptly transferred to a storage jar when still in the vacuum oven, then closed and sealed with Parafilm tape.

### 3.3. Liquid-Assisting Grinding (LAG) for Gemcitabine Encapsulation

The LAG was conducted by an automatic high-frequency grinder of model Retsch Qiagen TissueLyser (from Retsch GmbH & Co. KG, Haan, Germany). It was equipped with two 5 mL stainless steel grinding vials operating in parallel. In each grinding vial, a sample of 0.25 mmol Gem was mixed with 0.25 mmol compound **2**, and then the grinding fluid was added (0.3 mL isopropanol). Further, each vial contained one stainless steel grinding ball of 7 mm in diameter, and the grinding frequency was 30 Hz. The grinding time was set by repeating intervals: 5 min. ON + 5 min. OFF, so that the total grinding time was 60 min. This was done to avoid over-heating of the sample during the LAG. The obtained product (in the form of thick paste) was outgassed overnight in the vacuum desiccator equipped with a two-stage oil-free diaphragm vacuum pump and the manometer. The pumping speed was 50 L/m, and base pressure was 85 kPa below 1 atm. The obtained powder was termed “composite” and kept in a sealed specimen glass jar until use.

### 3.4. Instrumental Analysis of Samples

The ATR-FTIR spectra of samples (before and after LAG) were obtained by an infrared spectrometer model Nicolet iS20 (from Thermo Fisher Scientific, Madison, WI, USA). It was equipped with an ATR attachment of model Smart iTX (from Thermo Fisher Scientific). The spectra were collected using OMNIC software version 9, spectral resolution was 4 cm^−1^, the increment of the wavenumber was 0.5 cm^−1^, optical aperture was set to “Open”, the variable gain was used, and each spectrum was averaged 512 times. An attempt to use higher resolution at 2 cm^−1^ resulted in low absorbance, particularly within 3600–3000 cm^−1^ and poor spectral quality.

To eliminate effects of water vapor in air on IR spectra, the interior of the FTIR spectrometer was continuously purged with dried air at 30 scfh (standard cubic feet per hour) measured by flowmeter model RMA-7 (from Dwyer Instruments Inc., Michigan City, IN, USA). The dried air produced by an FT-IR Purge Gas Generator model 74-5041 Parker Balston (from Parker Hannifin Corporation, Haverhill, MA, USA) was of spectroscopic quality, with the remaining water vapor content equivalent to a dew point of −73 °C (or relative humidity RH < 1%). To continuously monitor the quality of FTIR spectra and remove artifacts due to trace water vapor, the OMNIC program had “Atmospheric Correction” parameter enabled and “Spectral Quality Results” parameter set at “H_2_O level” > 95%. The ATR-FTIR spectra were presented in absorbance mode.

Powder X-ray diffraction (XRD) patterns were obtained by diffractometer model MiniFlex (from Rigaku Corporation, Tokyo, Japan) equipped with nickel foil to filter out the K-beta artifact. Here, a Cu K-alpha line at 0.15418 nm was used, and the increments of the 2θ angle were of 0.02 deg. The numeric fitting of the ATR-FTIR and XRD peaks was conducted by Microcal Origin 2016 program.

### 3.5. Procedure of Delayed Drug Release to PBS

The tests were conducted using the automated dissolution tester model VK 7000 (from VanKel Industries, Edison, NJ, USA). It was equipped with the heater/circulator model VK 750D, peristaltic pump model VK-810 and automatic dissolution sampling station model VK 8000 (all from VanKel Industries). The overall procedure was similar to the one in [18]. Namely, the paddle method was employed, with stirring speed at 200 or 60 rpm (revolutions per minute). The dissolution medium was 750 mL of the 1X phosphate-buffered saline (PBS) without calcium and magnesium that was prepared by dilution of powder of PBS (from Albert Bioscience Inc., Laguna Hills, CA, USA) with DI water, followed by adjustment of pH to 7.4. The dissolution medium was maintained at 37 ± 0.5 °C in a one-liter glass dissolution vessel of the VK 7000 instrument, using its water thermostat bath and heater/circulator VK 750D. The sampling cannulas of the automated dissolution tester VK 7000 were equipped with 10-micrometer porous filters (UHMW polyethylene, product FIL010-01-a from Quality Lab Accessories, Telford, PA, USA) to avoid withdrawal of powder from drug release suspension and blockage of the dissolution sampling station model VK 8000.

The encapsulation matrix (compound **2**) actAl-MOF-TCPPH_2_ has Hill formula C_48_H_28_O_10_N_4_Al_2_ and formal molar mass 874 mg/mmol, while Gem has molar mass of 263 mg/mmol. For the equimolar amounts of these compounds in the composite, their initial mixture corresponded to the molar mass 874 + 263 = 1137 mg/mmol, and the weight content of Gem was 100% × 263/1137 = 23 wt.%.

In drug release experiments, specimens were used that contained 33 mg of Gem (for pure Gem) or the proportional amount of the composite. All experiments were conducted under sink conditions [30], namely the molar concentration of Gem in release medium (PBS) was always at least 3 times lower than the solubility of Gem in PBS.

The stirring of PBS was started in the dissolution vessel, and the calculated mass of sample was dropped into the dissolution medium. Then, at the predetermined time intervals (45 min. for the first 10 samplings, then 240 min. for the subsequent samplings), the 2 mL aliquots were automatically withdrawn and collected in the VK 8000 dissolution sampling station. The collected liquid samples were frozen at −80 °C until batch analysis by the HPLC-UV method.

### 3.6. Chromatographic Analysis of Released Gemcitabine

The collected samples were thawed and filtered, using PTFE syringe filters of 0.22 μm pore size and 4 mm diameter with Luer-Lok connectors (product SF17504 from Tisch Scientific, Cleves, OH, USA) and disposable 1 mL Luer Lock Tip Syringes (from BH Supplies, Jackson, NJ, USA). The concentration of Gem in each filtered sample was determined by the HPLC-UV method, using the instrument of series 1100 (from Agilent Technologies Inc., Santa Clara, CA, USA) and software ChemStation for LC 3D systems, version B.04.02. The analysis protocol was similar to that in [31]; namely, an isocratic mobile phase was a 25:75 *vol*/*vol* mixture of acetonitrile and 1.36% aqueous solution of ammonium acetate at 25 °C. A reverse phase HPLC column of model Eclipse XDB-C18 (4.6 × 150 mm, 5 μm, product 993967-902 from Agilent, Santa Clara, CA, USA) was equipped with guard cartridge (product 820950-925 from Agilent). The flow rate of the mobile phase of 0.5 mL/min, injection volume of 1 µL and detection wavelength of 254 nm were used. The calibration plot of Gem by the HPLC-UV analysis was prepared using the set of standard solutions of Gem in PBS.

### 3.7. Measurement of the Long-Term Cell Proliferation (Growth) by xCELLigence Instrument

PANC-1 cells (product CRL-1469 from ATCC, Manassas, VA, USA) were maintained at the Translational Core Facility of the University of Maryland Marlene and Stewart Greenebaum Cancer Center. The impedance-based real-time measurement of cellular proliferation (growth) was performed on the xCELLigence Real-Time Cell Analyzer (RTCA) in the designated 16-well electrode plates (E-plates; from Agilent) under standard culture conditions. The RTCA software version 1.2.1.1002 was used for data recording and analysis of proliferation. In all experiments, 100 μL of cell-free medium was added to each well of the E-plate, and background measurement was performed. Next, 100 μL of cell suspension (12,000 cells/100 μL) was added to each E-well, the measurement was started, and the cells were allowed to attach and proliferate for about 24 h. prior to addition of the cytotoxic compound. The readings were performed every 15 min. for up to 6 days (144 h). The readout was recorded by the RTCA system and was expressed as a dimensionless cell index (CI) which correlates with number and size of live cells. The measurements were performed in triplicates.

## 4. Conclusions

The encapsulation of Gem on the matrix of metal-organic framework Al-MOF-TCPPH_2_ (compound **2**) was successfully conducted using the novel and facile approach of LAG. This process results in a composite with chemical bonds between the Gem drug and the matrix; the interaction involves the C=O group of Gem and the formation of encapsulation complex Gem_x_MOF_y_. Further, the delayed release of Gem from the composite to PBS at 37 °C is demonstrated, using the automated drug dissolution apparatus with an autosampler, followed by the quantitative HPLC-UV analysis. The composite shows a delayed release of Gem up to 1800 min. and a constant concentration of Gem in the release media thereafter. In contrast, pure Gem shows an instant dissolution within <45 min. The delayed release of Gem from the composite follows the kinetic pseudo-first-order rate law. The kinetic rate constant *k* of delayed release decreases when stirring speed is decreased, which indicates diffusion control of drug release. The finding of in vitro delayed release of Gem from the composite to PBS is confirmed by the time-dependent inhibition of growth of PANC-1 cells in the longer time period up to 144 h, using the xCELLigence real-time cell analyzer. The delayed release of gemcitabine from the composite in this work is promising for the materials science of potential drug-eluting implants aimed at emerging local chemotherapy of solid tumors.

## Figures and Tables

**Table 1:** Assignments of IR spectral peaks of Gem in this work.

Wavenumber, cm^−1^	Assignment to Functional Group	Tautomer [22]
3477	*v*(N–H) in the imino-keto tautomer	imino-keto
3390	*v_sym_*(NH_2_)	amino-keto
1694	*v*(C=O)	imino-keto
1657 three shoulders	*v*(C=O)	imino-keto

## Data Availability

Data are contained within the article and Supplementary Materials.

## Supplementary Materials

The following supporting information can be downloaded at https://www.mdpi.com/article/10.3390/molecules29133189/s1, Figure S1: The simplified molecular structure of drug encapsulation matrix compound **2**. Figure S2: The ATR-FTIR spectra of the Gem drug. (a) High wavenumber range; (b) mid-IR range; (c) low wavenumber range. Figure S3: The ATR-FTIR spectra of matrix compound **2**. (a) High wavenumber range; (b) mid-IR range; (c) low wavenumber range. Figure S4: Powder XRD pattern of Gem before and after LAG. Figure S5: Powder XRD pattern of compound **2** before and after LAG. Figure S6: The ATR-FTIR spectra of Gem after LAG vs. composite (Gem + compound **2**) after LAG. (a) High wavenumber range; (b) mid-IR range; (c) low wavenumber range. Figure S7: (a) Powder XRD patterns of Gem after LAG and composite (Gem + compound **2**) after LAG; (b) peak fitting for Scherrer analysis of Gem after LAG; (c) similar fitting for composite (Gem + compound **2**) after LAG. Figure S8: Calibration plot of gemcitabine determined by HPLC-UV analysis.
